# Increased resting-state alpha coherence and impaired inhibition control in young smokers

**DOI:** 10.3389/fnins.2022.1026835

**Published:** 2022-11-09

**Authors:** Zhengxi Wang, Fang Dong, Yaning Sun, Juan Wang, Ming Zhang, Ting Xue, Yan Ren, Xiaoqi Lv, Kai Yuan, Dahua Yu

**Affiliations:** ^1^Inner Mongolia Key Laboratory of Pattern Recognition and Intelligent Image Processing, School of Information Engineering, Inner Mongolia University of Science and Technology, Baotou, China; ^2^College of Information Engineering, Inner Mongolia University of Technology, Hohhot, China; ^3^School of Life Science and Technology, Xidian University, Xi’an, China

**Keywords:** alpha coherence, resting state, young smokers, inhibition ability, Go/NoGo task

## Abstract

Exposure to nicotine is the first cause of entirely preventable death killing, which is commonly initiated in adolescence. Previous studies revealed the changes of electroencephalography (EEG) and inhibition control in smokers. However, little is known about the specific link between alpha coherence during the resting state and inhibition control ability in young smokers. The present study aimed to investigate inter-hemispherical and frontal-parietal alpha coherence changes and assessed the relationships between alpha coherence and inhibition control in young smokers. We collected resting-state EEG data from 23 young smokers and 24 healthy controls. Inhibition control ability was assessed by a Go/NoGo task. Compared to healthy controls, young smokers exhibited increased inter-hemispherical and frontal-parietal alpha coherence. Furthermore, young smokers committed more NoGo errors in the Go/NogGo task. It is noteworthy that alpha coherence at the frontal electrode sites was positively correlated with NoGo errors in healthy controls, whereas inverse correlations were observed in young smokers. Our findings suggested that alterations of alpha coherence may provide support to the earlier nicotine-dependence-related research findings, which may help us to understand the neuropathology of inhibitory control in young smokers.

## Introduction

Cigarette smoking is the first cause of entirely preventable death, which may kill approximately eight million people annually (World Health Organization [WHO], 2019). “Chinese Center for Disease Control and Prevention” indicated that the prevalence of cigarette smoking for students in junior high school was 10.6% for males and 1.8% for females.^1^ Furthermore, the brain will experience a series of significant physical, psychological, and social function development during the period from childhood to adulthood (Casey et al., 2005; Li et al., 2015). It is proposed that young adults are especially sensitive to exposure to drugs of abuse, such as smoking (Gulley and Juraska, 2013).

Smoking may have impact on cognitive control, particularly inhibition control (Yin et al., 2016). The inhibition control may be related to the ability to implement the inhibition of addictive behavior, which was demonstrated by previous electroencephalography (EEG) studies involving Go/NoGo tasks (Groman et al., 2009; Liu et al., 2019; Dong et al., 2021). The resting-state EEG provides a way of reflecting the intrinsic operation of corticocortical fiber systems (Murias et al., 2007b). Especially, EEG coherence, as a powerful tool for the diagnosis and treatment of diseases, provides information on the synchronization between different cortical regions at a high temporal resolution and reflects functional cortical connectivity at the centimeter scale (Bowyer, 2016; Bu et al., 2019). Higher EEG coherence value between two electrode pairs may reflect more intensive communication between different brain regions (Thatcher et al., 2005). Therefore, EEG coherence may offer significant advantages to investigate the mechanism of nicotine addiction in terms of high temporal resolution and measures of the brain network (Bu et al., 2019).

Several studies explored the EEG coherence basis of smoking and reported abnormal EEG coherence in smokers (Li et al., 2017; Bu et al., 2019). A previous study found that smokers exhibited increased values in overall EEG coherence than non-smoking controls during cue reactivity tasks (Bu et al., 2019). There is accumulating evidence that resting-state EEG coherence is a feasible method, which can measure the synchrony of brain activity across different brain regions, in the absence of task performance (Friston, 2011; Bu et al., 2019). The mechanism of reducing cigarette craving after hypnotic aversion suggestions was explored by using resting-state EEG coherence (Li et al., 2017). Furthermore, a finding provides evidence that large-scale communication between cortical regions may be modulated by alpha coherence (Chapeton et al., 2019). Inhibition control is commonly evaluated with Go/NoGo tasks, which can reflect the cognitive ability to withhold addiction behaviors. The inhibition control impairment has been implicated in difficulty to resist cigarettes, as demonstrated by more NoGo errors in the Go/NoGo tasks (Mashhoon et al., 2018). A study using meta-analysis approaches reported that inhibition control deficits were observed in smokers (Smith et al., 2014). However, few studies investigated the relationships between alpha coherence and inhibition control in young smokers.

In the current study, we investigated alpha coherence during the resting state and inhibition control by using the Go/NoGo tasks in young smokers and healthy controls. The relationships between alpha coherence and inhibition control performance were analyzed. Based on previous studies, we hypothesized that young smokers may make more NoGo errors with regard to the Go/NoGo task and exhibit increased alpha coherence relative to healthy controls.

## Materials and methods

### Participants

Twenty-three young smokers (mean age: 20.3 ± 0.7 years) and 24 healthy controls (mean age: 20.3 ± 0.8 years) were recruited from the Inner Mongolia University of Science and Technology through a smoking advertisement. Exclusion criteria included a history of neurological diseases, psychopathological illness, and substance abuse/dependence (excluding nicotine). All participants were right-handed as measured by the Edinburgh Handedness Questionnaire (Casey, 2015). Participants were classified according to the following criteria. For young smokers: (1) smoked >10 cigarettes daily for the last half a year, (2) reported nicotine dependence according to DSM-V criteria, (3) monoxide (CO) concentrations >6 ppm, and (4) no period of smoking cessation longer than 3 months in the past few years (Dong et al., 2021). In addition, we administered the Fagerström Test for Nicotine Dependence (FTND) to assess nicotine dependence (Fagerstrom and Schneider, 1989). For healthy controls: (1) had consumed < cigarettes in their lifetime, and (2) their parents and roommates had not smoked (to avoid the effects of second-hand smoke) (Dong et al., 2021). The demographic characteristics and smoking status of participants were presented in Table 1.

**TABLE 1:** Demographic characteristics of the participants.

Demographic variables	Smokers (*n* = 23)	Controls (*n* = 24)	*P*-value
Age (years)	20.3 ± 0.7	20.3 ± 0.8	0.7
Age range (years)	19–21	19–21	–
Years of education (years)	14.0 ± 0.7	14.2 ± 0.8	0.25
Cigarettes per day (CPD)	14.4 ± 5.5	–	–
Age of smoking onset	14.7 ± 3.2	–	–
Duration of smoking	4.4 ± 2.5	–	–
Pack-Years	3.3 ± 2.9	–	–
FTND score	4.6 ± 1.7	–	–

Values are expressed as means ± standard deviations. Pack-years: Duration of smoking × CPD/20. FTND, Fagerström test for nicotine dependence.

### Procedure

After receiving an explanation of the experiment, all participants signed an informed assent. Previous studies have reported that a single 21 mg dose of transdermal nicotine may exert influence on EEG activation (Knott et al., 1999; Knott, 2001). In order to minimize the acute effects of nicotine, young smokers were required to refrain from smoking about 1 h before the start of the experiment (Yin et al., 2016). Then participants were seated in a comfortable environment with dimly lit and electrically shielded. Inhibition control was generally assessed by using Go/NoGo tasks, in which participants were instructed to respond as quickly and accurately as possible to frequent Go stimuli (88.4% probability) but to withhold responses to infrequent NoGo stimuli (11.6% probability) (Littel et al., 2012). The Go/NoGo task consisted of four blocks involving 159 capital letters (e.g., A, B, C, and D), with 60 s of rest after each block (Liu et al., 2019). The duration of each letter was 700 ms, and the interval was 300 ms (Dong et al., 2021). A total of 10-min resting-state EEG data were recorded with participants’ eyes closed and comfortably seated. The resting-state EEG signals from 3 to 8 min in the middle of the data were selected.

### Electroencephalography recording and data analysis

A digital BrainAmp MR plus amplifier (Brain Products GmbH. Munich. Germany) was used for EEG recording in the current study. EEG signals were recorded at a sampling rate of 1,000 Hz with a filter of 0.10–250 Hz, using 64 Ag/AgCl electrodes mounted on different scalp sites according to the modified 10/20 International System. Horizontal electrooculogram (EOG) signals were acquired with electrodes placed above the left eye, and vertical EOG signals were recorded with electrodes located by the side of the external canthus of the right eye. The impedance values of all recording electrodes were kept under 5 kΩ. Offline processing was performed with EEGLAB (Delorme and Makeig, 2004) and customized MATLAB code. EEG data were filtered in the temporal domain using a band-pass filter of 0.1–50 Hz (with a 50 Hz notch filter). Some visible artifacts, such as eye movements and eye blinks, were removed using an ICA method. Subsequently, the 5 min data were segmented into 2 s epochs (500 sample points per epoch) which were visually inspected to remove physical artifacts, re-referenced to the average reference, and down-sampled to 250 Hz.

It should be remarked that EEG coherence measured the synchrony of brain activity across different brain areas (Murias et al., 2007a,b), which was used in substance-dependence-related studies (Tcheslavski and Gonen, 2012; Lee et al., 2019). To obtain EEG coherence between two electrodes at each frequency, the average cross spectrum, which was calculated with the complex conjugate of Fourier coefficients, should be squared and normalized by the average residual power spectrum (Fagerstrom and Schneider, 1989; Srinivasan et al., 2007). Coherence in alpha was performed by averaging coherence values in corresponding frequency interval (Bu et al., 2019). Power at underlying sources may have effects on coherence measurements at short (<5 cm) interelectrode distances (Srinivasan et al., 1998; Murias et al., 2007b). Accordingly, we may not statistically distinguish young smokers at short interelectrode distances. Inter-hemispherical and frontal-parietal coherence changes appear to reflect genuine group differences in neuronal activity, since the distances between those electrode pairs were larger than 5 cm (Babiloni et al., 2006).

The present study investigated the inter-hemispherical and frontal-parietal coherence of alpha rhythm in young smokers and healthy controls. In order to explore inter-hemispherical and frontal-parietal alpha coherence at electrode pairs (Babiloni et al., 2006), the following eight channels were selected to generate resting-state EEG coherence values: F3, F4, C3, C4, P3, P4, Fz, Pz. Then, alpha coherence values were extracted. Three inter-hemispherical coherence values were calculated between the frontal (F3–F4), central (C3–C4), and parietal (P3–P4) electrode pairs. Frontal-parietal coherence values were examined using the following pairs of electrodes: F3–P3, Fz–Pz, and F4–P4. A cortical map of inter-hemispherical and frontal-parietal alpha coherence was presented using the BrainNet Viewer for MATLAB (Xia et al., 2013).

### Statistical analysis

Statistical analyses were performed using SPSS version 24 software (SPSS Statistics, IBM, Armonk, NY, USA). Statistical data, such as behavioral outcomes, demographics, and alpha coherence, were compared between young smokers and healthy controls using two independent samples *t*-tests. Furthermore, alpha coherence comparisons were corrected using false discovery rate (FDR) correction. Finally, to evaluate the relationships between resting-state alpha coherence and inhibition control in young smokers and healthy controls, partial correlation tests were used for the statistical analyses. Age was included in the analyses as a covariate. For all statistical analyses, the significance level was set at *p* < 0.05.

## Results

### Demographic and behavioral data

No significant changes in age and education were found between young smokers and healthy controls (Table 1). Significant group differences were observed in Go/NoGo tasks, showing that young smokers made more NoGo errors than healthy controls (young smokers: 29.70 ± 9.61, healthy controls: 21.04 ± 10.31, *p* = 0.005). Furthermore, there were no significant changes in Go reaction time (young smokers: 382.69 ± 47.40, healthy controls: 417.85 ± 88.34, *p* = 0.098) between the two groups.

### Alpha coherence

**FIGURE 1:**
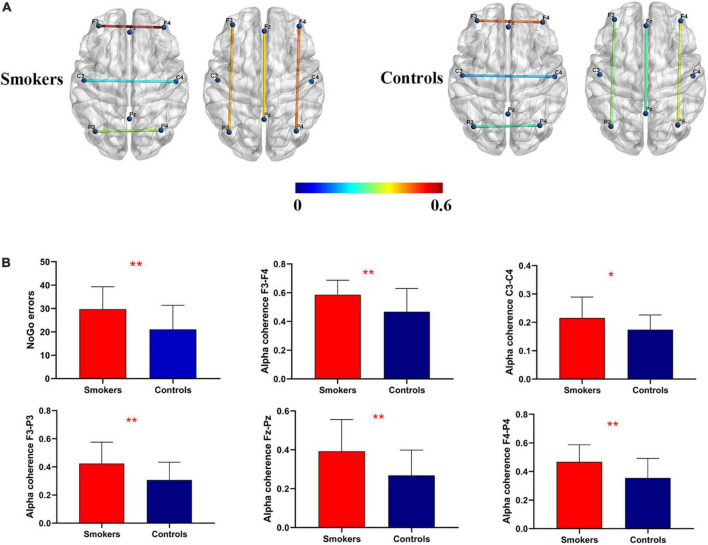
Coherence mapping view and increased alpha coherence. **(A)** Cortical representation of the inter-hemispherical and frontal-parietal alpha coherence in young smokers (left) compared with healthy controls (right). **(B)** More NoGo errors and increased alpha coherence at C3–C4, F3–F4, F3–P3, F4–P4, and Fz–Pz electrode pairs were found in young smokers compared with healthy controls (*p* < 0.05).

For the inter-hemispherical coherence analysis, there were significant changes in alpha coherence between the groups. Young smokers exhibited increased alpha coherence values than healthy controls across the frontal (F3–F4) (*F* = 3.427, *p* = 0.009) and the central (C3–C4) (*F* = 0.283, *p* = 0.0372) electrode pairs. Similarly, there were significant changes in alpha coherence with regards to frontal-parietal coherence. Young smokers demonstrated increased alpha coherence values across the left frontal-parietal (F3–P3) (*F* = 1.226, *p* < 0.01), the midline frontal-parietal (Fz–Pz) (*F* = 3.474, *p* < 0.01), and the right frontal-parietal (F4–P4) (*F* = 0.968, *p* < 0.01) electrode pairs (Figure 1).

### Correlation analyses

**FIGURE 2:**
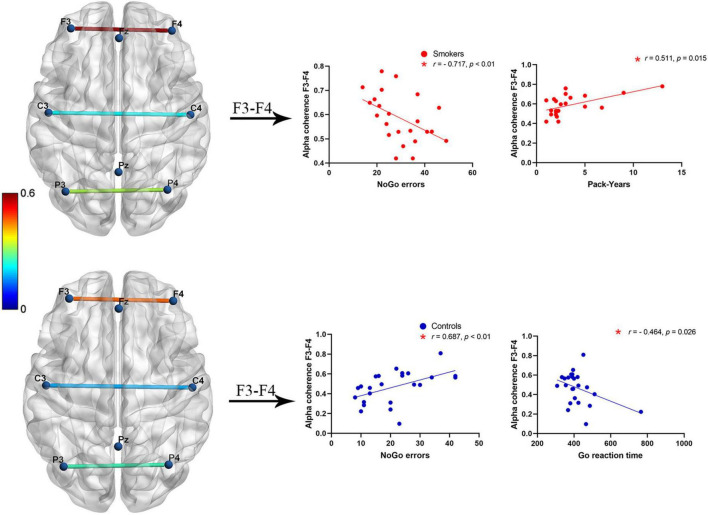
A negative correlation between alpha coherence at F3–F4 electrode pairs and NoGo error numbers was found in young smokers (*p* < 0.01), whereas a positive correlation between alpha coherence at F3–F4 electrode pairs and NoGo error numbers was found in healthy controls (*p* < 0.01). Inverse patterns of correlations between alpha coherence at F3–F4 electrodes and Go reaction time (*p* < 0.05). Positive correlations between Pack-Years and F3–F4 alpha coherence in young smokers (*p* < 0.05).

Partial correlation tests were used for resting-state alpha coherence, behavioral data of the Go/NoGo task, and smoking status. Significant positive relationships between NoGo error numbers and alpha coherence across F3–F4 electrode pairs were found in healthy controls (*r* = 0.687, *p* < 0.01). In contrast, significant negative relationships between NoGo error numbers and F3–F4 alpha coherence were identified in young smokers (*r* = −0.717, *p* < 0.01). A significant correlation between Go reaction time and alpha coherence at the frontal (F3–F4) electrode pairs was identified in healthy controls (*r* = −0.464, *p* = 0.026). Furthermore, a positive correlation between Pack-Years and F3–F4 alpha coherence was observed in young smokers (*r* = 0.511, *p* = 0.015) (Figure 2).

## Discussion

In the present study, we investigated abnormalities of EEG alpha coherence during a resting state and inhibition control performance by using the Go/NoGo task. To begin with, we found that more NoGo response errors were observed in young smokers compared to healthy controls. Then, relative to healthy controls, inter-hemispherical alpha coherence values across the frontal (F3–F4) and the central (C3–C4) electrode pairs were found to be increased in young smokers. Furthermore, young smokers exhibited increased alpha coherence values across the frontal-parietal (F3–P3, Fz–Pz, and F4–P4) electrode pairs than healthy controls. Finally, our analyses revealed significant relationships between inter-hemispherical alpha coherence across the frontal (F3–F4) electrode pairs and inhibition control capacity.

The current study reported that increased NoGo response errors were found in young smokers than healthy controls, which was consistent with previous studies (Luijten et al., 2011; Nestor et al., 2011). It was demonstrated that NoGo response errors may reflect the inhibition ability (Luijten et al., 2011). Nicotine intake during adolescence might make an impact on sensitive cognitive developmental processes, thus contributing to poor performance in the Go/NoGo task (Counotte et al., 2009; Goriounova and Mansvelder, 2012). A finding suggested that early onset smokers failed more frequently to Go response accuracy errors and made more errors with regards to NoGo trails than late onset smokers (Mashhoon et al., 2018).

In the present study, we detected increased alpha coherence across F3–F4, C3–C4, F3–P3, Fz–Pz, and F4–P4 electrode pairs during a resting state in young smokers. Previous EEG studies reported consistent results for smokers, such as increased inter-hemispherical alpha coherence values for young and elderly adult smokers when compared to age-matched healthy controls (Knott and Harr, 1997). Alpha coherence under the resting state revealed the neural changes associated with smoking, such that current smokers and past smokers showed higher alpha coherence than non-smokers (Lee et al., 2022). In addition to these results, the previous studies reported that altered EEG coherence was found in individuals with substance dependence [such as nicotine (Li et al., 2017), alcohol (Winterer et al., 2003), and heroin (Franken et al., 2004)]. These results showed that EEG coherence may be considered as a support to the earlier substance-related research findings.

In this study, we evaluated associations between alpha coherence and task performance in young smokers and healthy controls. Our findings showed that alpha coherence across the frontal (F3–F4) electrode pairs was inversely correlated with NoGo errors in young smokers, while a positive correlation between alpha coherence at F3–F4 electrode pairs and NoGo errors was observed in healthy controls. The relationship between alpha coherence and Go reaction time was identified in healthy controls. Furthermore, we detected a positive correlation between alpha coherence and Pack-Years in young smokers. All significant correlations we identified would be helpful to establish a link between alpha coherence and impaired inhibition control in young smokers. Relationships between EEG signals and impaired inhibition control were reported by several studies. A recent study revealed reduced NoGo-N2 amplitudes in smoking groups and suggested that NoGo-N2 amplitudes were associated with inhibition ability (Nieuwenhuis et al., 2003; Falkenstein, 2006; Luijten et al., 2011). The brain was continuously active, and EEG alpha rhythms persisted even during a resting state (Mantini et al., 2007). There is considerable evidence indicating that alpha oscillations, as a mechanism for cerebral integration, may be related to inhibitory functions (Knyazev, 2007). A study showed that increased alpha local oscillations were believed to be vital for inhibitory motor control (Hummel et al., 2002). Multimodal studies reported that activation of the resting-state network (RSN) was similar to EEG alpha rhythms (Goldman et al., 2002; Mantini et al., 2007). Our current findings indicated that alpha coherence was linked with inhibition control processes. These correlation analyses showed that resting-state alpha coherence at the frontal (F3–F4) electrode pairs might exert influence on inhibition control ability in a meaningful manner. These findings suggested that altered EEG alpha coherence may be characteristic of inhibition control performance. Alterations of alpha coherence values may provide support to the earlier nicotine-dependence-related research findings.

## Limitation

There were several limitations in the present work. Firstly, the sample size of our study was relatively small to explore the pathology of inhibitory control in young smokers. Secondly, the study focused only on male smokers. Future studies should aim to increase sample size and improve complex designs; these strategies will help us to fully explore smoking mechanisms in young smokers.

## Conclusion

The current study suggested that young smokers exhibited more NoGo errors and showed inhibition control impairments. Furthermore, young smokers exhibited increased alpha coherence at the frontal, central, left frontal-parietal, midline frontal-parietal, and right frontal-parietal electrode pairs. Importantly, alpha coherence at the frontal electrode pairs was correlated with NoGo errors in both young smokers and healthy controls. The study focused on the relationship between alpha coherence and inhibition control and may provide novel insights into the treatment of nicotine in young smokers.

## Data availability statement

The original contributions presented in this study are included in the article/supplementary material, further inquiries can be directed to the corresponding authors.

## Ethics statement

The studies involving human participants were reviewed and approved by the Medical Ethics Committee of the First Affiliated Hospital of Baotou Medical College, Inner Mongolia University of Science and Technology. The patients/participants provided their written informed consent to participate in this study. Written informed consent was obtained from the individual(s) for the publication of any potentially identifiable images or data included in this article.

## Author contributions

DY, KY, and ZW designed the experiments. ZW and YS performed the experiments. ZW and FD analyzed the data and wrote the manuscript. MZ, TX, YR, KY, and DY provided the critical revision of the manuscript. All authors critically reviewed the content and approved the final version for publication.

## Footnotes

1
http://www.chinacdc.cn/
